# High Dose Rate (HDR) Brachytherapy for Mycosis Fungoides of the Wrist

**DOI:** 10.4236/ijcm.2015.63020

**Published:** 2015-03-19

**Authors:** Gaurav Shukla, Virginia Lockamy, James Keller, Joya Sahu, Barbara Pro, Onder Alpdogan, Wenyin Shi

**Affiliations:** 1 Department of Radiation Oncology, Thomas Jefferson University, Philadelphia, PA, USA; 2 Department of Dermatology, Thomas Jefferson University, Philadelphia, PA, USA; 3 Department of Medical Oncology, Thomas Jefferson University, Philadelphia, PA, USA

**Keywords:** Mycosis Fungoides, Cutaneous T-Cell Lymphoma, Radiation Therapy, High Dose Rate (HDR)

## Abstract

Mycosis fungoides (MF) is the most common cutaneous T-cell lymphoma accounting for approximately half of all cutaneous T-cell lymphomas. Radiation therapy is an effective treatment for early stage MF and has been shown to result in long-term disease-free intervals, with even curative potential. Radiation is also effective as palliative treatment for the localized lesion resistant to the topic or other treatments. In the current study, we report using high dose rate (HDR) radiation treatment for a patient with resistant mycosis fungoides involving the wrist. We report a convenient treatment with an ideal radiation dose distribution, and a excellent clinical outcome.

## 1. Introduction

Mycosis fungoides (MF) is the most common cutaneous T-cell lymphoma accounting for approximately half of all cutaneous T-cell lymphomas [1]. It typically presents with pruritic macular or patchlike scaled lesions and progresses to formation of erythematous plaques which can cover any percentage of the skin, depending on the severity. Most patients with MF are treated with skin-directed therapies, with or without systemic treatment. Radiation therapy is an effective treatment for early stage MF and has been shown to result in long-term disease-free intervals, with even curative potential [2] [3]. Electron beam therapy is most commonly used, since electrons of appropriate energy can be used to confine the dose of radiation to the superficial layers of the skin to avoid damage to deeper tissues. However, there are some limitations of electron beam radiation. These include poor dose uniformity while treating sloped or irregular skin surfaces, as well as hot spots and cold spots at junctions when matching fields are used. When a large circumference of a limb is involved, multiple matching electron fields would be needed, resulting in significant dose heterogeneity, particularly at match lines. Photon treatment in this setting results in treating the entire limb, increasing the risk of lymphedema. In an attempt to achieve better dose homogeneity to the involved area, while minimizing deep tissue irradiation, we describe a HDR brachytherapy technique using a Freiburg flap [4].

## 2. Case Presentation

Our patient, a 64-year-old female, presented with a long history of eczema. Multiple biopsies of an enlarging, weeping, pruritic forearm lesion were negative for MF, but a clinical diagnosis of MF was eventually made by dermatologists and medical oncologists caring for the patient. Staging workup, including PET and peripheral blood smear, were negative for visceral or hematogenous involvement. She was initially treated with topical therapy, including clobetasol, and phototherapy, but due to her intolerance of these treatments and their poor efficacy, she was referred for consideration of radiation therapy.

**Figure 1.**
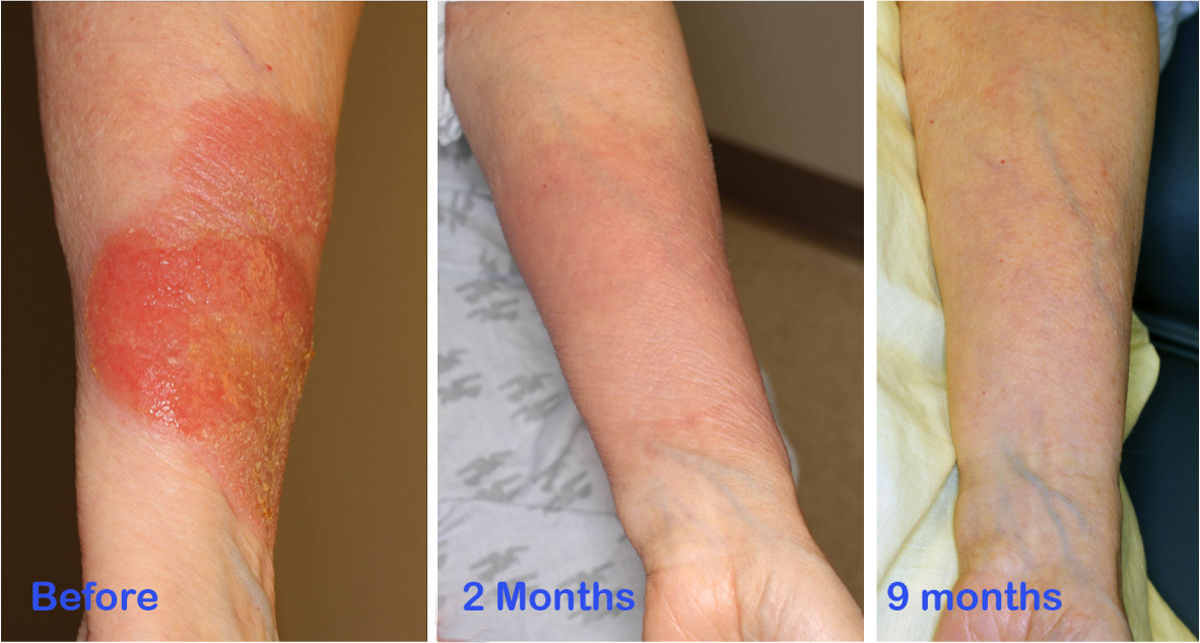
On the left, the pre-treatment area of disease over the forearm. The lesion extended 270° around the circumference of the arm. In the middle, the post-treatment imaging taken 2 months after treatment, with Grade 1 dermatitis as the lone residual side effect. On the right, the post-treatment imaging taken 9 nine months after treatment, with complete response and excellent cosmetic outcome.

On presentation, she had a nearly circumferential erythematous and raised plaque on her forearm (Figure 1), which had remained pruritic and had some scaling and crusting. In view of the circumferential nature of the lesion, we proposed a brachytherapy technique. The recent development of the Freiburg Flap (Elekta, Stockholm, Sweden), which consists of a flexible mesh style surface mold to which HDR cathethers can be connected, facilitates homogenous coverage of curved, superficial target volumes that are otherwise difficult to treat. We adapted a Freiburg flap to treat our patient’s localized MF lesion.

**Figure 2.**
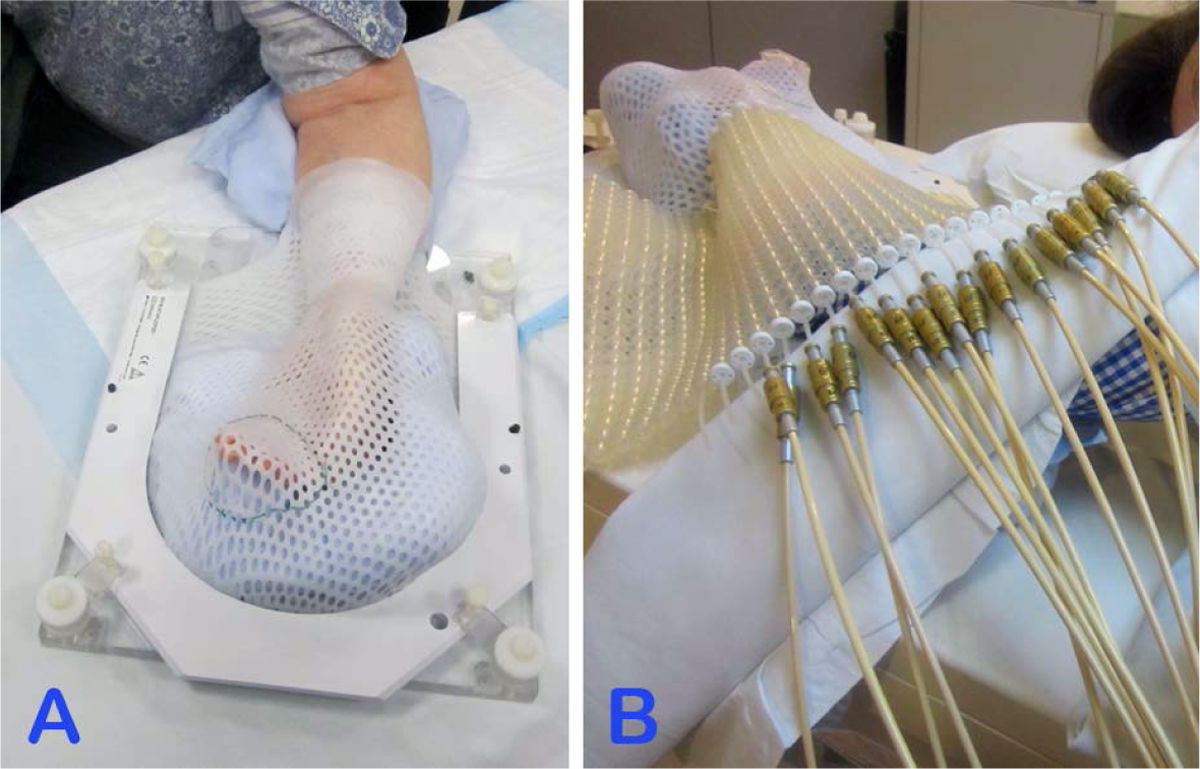
A custom accuform immobilization device was used to immobilize the patient’s arm, with aquaplast placed over the top (A). A Freibug flap was attached to the aquaplast with markers placed in every other catheter (B), and the patient was placed in the prone position for simulation and treatment.

**Figure 3.**
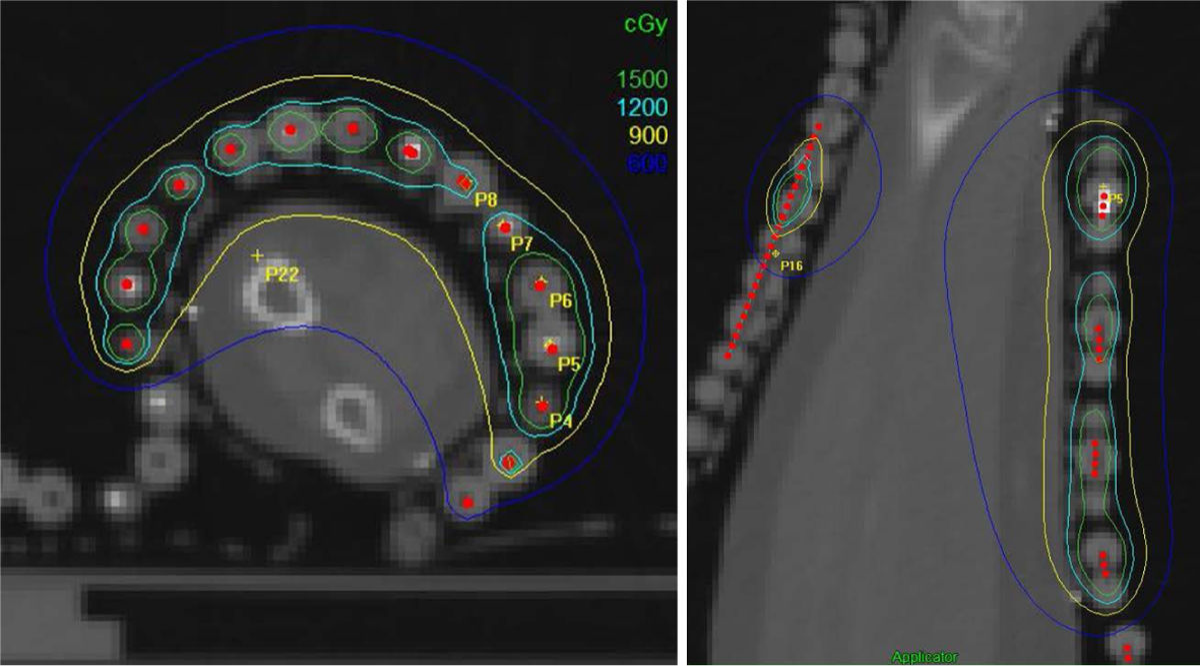
Isodose lines with the total dose of 9 Gy reaching a depth of 3 mm below the skin surface.

The patient left arm was immobilized in a custom accuform immobilization device, with aquaplast on the top. A Freibug flap was attached to the aquaplast with markers placed in every other catheter (Figure 2). The lesion borders were wired for visualization on the simulation scan. The patient underwent CT simulation in treatment position. Planning target volumes and the catheters were contoured for dosimetric planning purposes. A total of 16 catheters in the flap were used. The total dose was 9 Gy in 3-Gy fractions, prescribed to a depth of 3mm, which represented the lesion thickness (Figure 3). A conebeam CT was performed before each treatment to verify placement and position.

The patient tolerated the treatments well. Her post-treatment course was complicated by cellulitis, which responded to a course of oral antibiotics. She also described mild radiation-induced neuropathy that was self-limiting. Both of these symptoms had resolved at the time point of two months post treatment, with complete response of the lesion along with only Grade 1 dermatitis in the treatment field. She continued to have complete response with excellent cosmetic outcome at 9-month follow up with no toxicity (Figure 1). We will continue to follow the patient in a multi-disciplinary setting, with our dermatology service and medical oncology service also participating in management, to determine the duration of her response and the need for further treatment.

## 3. Discussion

Mycosis fungoides typically involves skin with a superficial depth, and thus electron therapy with limited penetration is often used. MF is highly sensitive to radiation treatment. Local radiation treatment can result in complete disease clearance in most patients. A dose of 15 – 25 Gy administrated over 1 – 3 weeks is often used. Complete response is seen in greater than 90% of the cases for the MF lesions receiving more than 3 Gy [5]. Resolution of the skin lesions usually happens quickly, typically by 2 – 3 weeks. Recent literature on low dose palliative radiotherapy showed a 92% response rate in localized disease treatment regimens involving superficial dosing as low as 8 Gy in two fractions [6]. A single fraction regimen also has been tested, and a dose of 7–8 Gy in one fraction resulted in over 90% complete response [7].

Though electron radiation may serve as an excellent option for the majority of patients, it has its limitations, particularly for sloped or curved anatomy, irregular surfaces, and large areas of coverage requiring matching fields. Standard x-ray (photon) radiation has a favorable dose distribution for sloped or irregular surfaces; however, it also delivers a higher dose to deeper normal tissues. It should be avoided when treating near critical deep structures, or when treating the entire circumference of a limb, to minimize the risk of lymphedema. In the current case, due to the location and circumferential nature of the lesion, neither electrons nor a conventional photon-based plan could achieve an ideal dose distribution. With the HDR technique described here, we were able to reduce the radiation dose to the deep normal tissue, avoiding treating the entire circumference, and achieve uniform dose coverage of the target lesion. The treatment delivery time is less than five minutes. This appears to be an excellent alternative to electron and photon beam for difficult locations. The delivery technique using a Freiburg flap is novel, and our initial success suggests that we may be able to treat similarly-presenting lesions using this technique.
